# Colorectal cancer promoter methylation alteration affects the expression of glutamate ionotropic receptor AMPA type subunit 4 alternative isoforms potentially relevant in colon tissue

**DOI:** 10.1007/s13577-021-00640-x

**Published:** 2021-10-30

**Authors:** Ana Florencia Vega-Benedetti, Eleonora Loi, Loredana Moi, Angelo Restivo, Francesco Cabras, Simona Deidda, Andrea Pretta, Pina Ziranu, Sandra Orrù, Mario Scartozzi, Luigi Zorcolo, Patrizia Zavattari

**Affiliations:** 1 Department of Biomedical Sciences, Unit of Biology and Genetics, University of Cagliari, Cittadella Universitaria di Monserrato, Cagliari, Italy; 2 Department of Surgery, Colorectal Surgery Center, University of Cagliari, Cagliari, Italy; 3 Department of Medical Oncology, University Hospital of Cagliari, Cagliari, Italy; 4 Department of Pathology, “A. Businco” Oncologic Hospital, ASL Cagliari, Cagliari, Italy

**Keywords:** Colorectal cancer (CRC), DNA methylation alterations, *GRIA4*, Gene expression, Gene regulation

## Abstract

DNA methylation alterations are early events during tumourigenesis, affecting genes involved in the crosstalk between cells and surroundings in colorectal cancer (CRC). Among these genes, *GRIA4*, Glutamate Ionotropic Receptor AMPA Type Subunit 4, displays hypermethylation in the promoter region, and is an early diagnostic biomarker. It is well known that methylation can also affect alternative transcription. The purpose of this study is to evaluate the expression, at transcript and protein level, of *GRIA4* main isoforms (the canonical one and a short variant) in 23 CRC and matched normal samples, of which we previously verified the methylation status. We further predicted miRNA/transcript target interactions as a possible post-transcriptional regulation using bioinformatics tools. As expected, downregulation of both variants has been observed in tumours. Interestingly, in contrast to what observed at transcriptional level, the GluR4 protein short isoform displayed higher expression than the canonical one either in normal or tumoural tissues. This may be explained by miRNA specifically targeting the canonical isoform. Our study is the first one that shows the expression of both isoforms in colon tissues. To note, the evident expression of the short isoform suggests a functional role in intestinal cell biology.

## Introduction

Cancer cells are characterized by massive changes in gene expression profile in terms of transcripts levels and expression of alternative isoforms, due to different biological mechanisms, among which alterations in DNA methylation patterns have a pivotal role [1–8].

DNA methylation at cytosines predominantly occurs in CpG dinucleotides known as CpG sites. Regions with high density of CpG sites, defined as CpG islands (CGIs), may overlap with transcription start sites. Alterations in DNA methylation pattern maintenance or de novo DNA methylation events can be associated with pathologies development. In particular, DNA methylation dramatically changes in cancer cells, i.e. a wide loss of DNA methylation occurs, whereas promoter-associated CGIs, usually un-methylated, undergo de novo methylation [4]. These aberrations, which can be cancer-specific, are considered early and frequent events in tumourigenesis, sometimes detected in premalignant tissues, becoming promising diagnostic biomarkers [9]. Promoter hypermethylation is frequently associated with gene silencing and can also affect the expression of alternative transcripts [6, 10]. Furthermore, DNA methylation alterations can be traced in cell-free circulating tumour DNA through non-invasive techniques [1, 3, 11, 12].

Other epigenetic alterations frequently found in tumours regard the post-transcriptional regulation. It has been reported abnormal expression of micro RNAs (miRNAs) that bind to target mRNAs blocking their translation and long non coding RNAs (lncRNAs) known as miRNAs sponges which antagonize miRNAs function [12–14].

In a genome-wide methylation study, we identified 74 CGIs significantly aberrantly methylated shared between colorectal cancer (CRC) and adenoma samples. Most of the genes associated with these altered CGIs encode for proteins essential for the interaction among cells and with their surrounding environment [1]. We evaluated the expression levels, using qRT-PCR and Western blot, of selected genes, barely expressed in normal colon, whose promoter CGIs were hypermethylated in CRC and we detected their further downregulation in tumour samples [1, 3]. Among these alterations, the CGI associated with *GRIA4* (Glutamate Ionotropic Receptor AMPA Type Subunit 4) gene reached very high methylation levels in tumour samples compared with normal ones and it has been shown as an excellent putative biomarker but also potentially functionally involved in CRC. This altered CGI is located in the promoter region of *GRIA4*, which encodes for two isoforms recognized by the same antibody, whose expression may be potentially affected by this alteration. Only the expression of the canonical isoform was evaluated in tumour and normal samples in our previous study [3]. *GRIA4* encodes a subunit of the AMPA tetrameric receptor complex. Each subunit consists of the extracellular amino-terminal domain, the extracellular ligand-binding domain, three transmembrane helixes plus a membrane re-entrant loop, and an intracellular carboxyl-terminal domain [15]. The principal function of this receptor type, as a cationic ion channel, is mainly performed in the central nervous system, e.g. synaptic communication [16]. *GRIA4* is included within the genes encoding for proteins that are involved in cell signalling and cross-talking, pathways frequently altered in cancer. Several transcript variants of *GRIA4* have been identified and annotated in Ensembl Genome browser (GRCh38.p13) (https://www.ensembl.org), including 11 protein coding transcripts of which two (ENST00000282499 and ENST00000393125) have both HAVANA and Ensembl gene annotation. Indeed, two protein isoforms have been described by the NIH full-length cDNA project, whereas other five potential ones have been computationally mapped [17] (https://uniprot.org). Regarding the first two isoforms, the shorter one consists of 433 amino acids but its function is not elucidated, whereas the longer isoform, 902 amino acids-length, is considered the canonical one. Interestingly, the structure of the shorter protein only comprises the extracellular domain of the receptor and it also differs from the canonical isoform in ten amino acids located at the C-terminal sequence.

Given the potential different functional roles of these isoforms in CRC, this work aims to investigate the expression of both isoforms at mRNA and protein levels, in tumoural and normal colon tissues.

## Materials and methods

### Tissue samples

The investigation cohort consisted of 23 CRC and their matched normal tissue samples collected from the Department of General Surgery of the University of Cagliari (Italy). Normal samples were taken at a distance > 10 cm from the neoplastic tissue. Clinical data are presented in Table 1.

**Table 1:** Clinical characteristics of CRC patients

Sample id	Tumour location	Stage at diagnosis	Mucinous histology	Lymphovascular invasion	Grade	Ulcerative neoplasia	Assays
CRC_2	Left colon	I	NO	NO	G2	NO	Methylation, transcript, protein
CRC_3	Right colon	III	NO	YES	G2	YES	Methylation, transcript, protein
CRC_8	Rectum	IV	YES	YES	G2	NO	Methylation, transcript, protein
CRC_11	Rectum	IV	NO	YES	G2	YES	Methylation, transcript
CRC_12	Right colon	0	NO	YES	G1	NO	Methylation, transcript, protein
CRC_14	Rectum	III	YES	YES	G3	NO	Methylation, transcript, protein
CRC_15	Left colon	0	NO	NO	G2	NO	Methylation, transcript
CRC_16	Left colon	IV	NO	YES	G3	NO	Methylation, transcript
CRC_18	Rectum	0	NO	NA	G2	NO	Methylation, transcript
CRC_19	Right colon	II	YES	YES	G2	YES	Methylation, transcript, protein
CRC_21	Transversal colon	II	NO	YES	G2	NO	Methylation, protein
CRC_25	Left colon	III	NO	YES	G2	NA	Methylation, transcript
CRC_29	Right colon	II	NO	YES	G2	NA	Methylation, transcript, protein
CRC_33	Right colon	IV	NO	YES	G2	NA	Transcript, protein
CRC_34	Rectum	III	NO	YES	G2	YES	Methylation, transcript, protein
CRC_38	Rectum	III	NO	YES	G2	NO	Methylation, transcript
CRC_41	Rectum	III	NO	YES	G2	NO	Methylation, transcript
CRC_42	Rectum	III	YES	YES	G3	YES	Methylation, transcript
CRC_45	Right colon	NA	NA	NA	NA	NO	Methylation
CRC_50	Transversal colon	II	NO	NO	G2	NO	Methylation, transcript
CRC_53	Left colon	II	NO	NO	G1	YES	Methylation, transcript
CRC_89	Right colon	I	YES	YES	G2	YES	Methylation, transcript
CRC_102	Rectum	III	NO	YES	G2	YES	Methylation, transcript

### DNA methylation analysis

Genomic DNA was extracted from tumoural and matched non-tumoural tissues using the DNeasy Blood & Tissue Kit (Qiagen, Hilden, Germany). DNA quantity of all samples was evaluated by spectrophotometric reading.

DNA samples were bisulfite converted using EZ DNA Methylation Gold Kit (Zymo Research, Irvine, CA, USA) following the manufacturer’s instructions.

*GRIA4* methylation analysis was performed by MethyLight qPCR as previously described in [3]. The methylation independent control reaction (*ALU-C4*) was used to normalize the quantity of DNA input [18]. The reaction contained: 1 × TaqMan Genotyping Master mix (Applied Biosystems, Foster City, CA, USA), 900 nM of each primer, 250 nM of probe, 50 ng bisulfite converted DNA in a final volume of 30 μl. Primers and probes sequences are reported in Table 2. Every reaction was performed in triplicate and the experiment was conducted on a DNA Engine Opticon 2 Real-Time Cycler (Bio-Rad, Hercules, CA, USA) using the following thermal conditions: initial PCR activation step at 95 °C for 10 min (min), followed by 50 cycles of denaturation step at 95 °C for 15 s (sec) and annealing/extension step at 60 °C for 1 min. *GRIA4* methylation levels were quantified using the ΔΔCt method [19]. ΔCt was calculated as the difference between Ct of the target assay and Ct of the *ALU-C4* control. Average ΔCts were calculated for tumoural and normal samples. ΔΔCt was calculated as the difference between the average tumour ΔCt and the average normal ΔCt. Samples showing Ct values higher than 45 were excluded.

**Table 2:** Primers and probes sequences for MethyLight assay

Target	Forward primer (5′–3′)	Reverse primer (5′–3′)	Probe (5′–3′)
*GRIA4*	GGGTTGGTGTAGGTTTGTT	CTCCCCCCTTACTTTCTCACATACACACAA	AACGCCGCGACCGCCACAC
*ALU-C4*	GGTTAGGTATAGTGGTTTATATTTGTAATTTTAGAT	ATTAACTAAACTAATCTTAAACTCCTAACCTCA	CCTACCTTAACCTCCC

### mRNA expression analysis

Total RNA was extracted from tumoural and normal samples using the RNeasy Mini Kit (Qiagen, Hilden, Germany) following manufacturer’s instructions. Reverse transcription of 1 μg of RNA to cDNA was performed using the High Capacity Kit (Applied Biosystems, Carlsbad, CA, USA). Gene expression was evaluated by qPCR using SsoAdvanced Universal SYBR® Green Supermix (Bio-Rad, Hercules, CA, USA). Primer sequences of *GRIA4* transcript variants, ENST00000282499 and ENST00000393125 defined as long and short respectively, and of the endogenous gene *TFRC* are reported in Table 3. PCR conditions were as follow: first step of denaturation at 95 °C for 2 min, followed by 50 cycles of denaturation at 95 °C for 15 s and annealing and extension at 60 °C for 1 min.

**Table 3:** Primers for qRT-PCR expression assay

Gene	Forward primer (5′–3′)	Reverse primer (5–3′)
*GRIA4 long*	CAAAGGCTATGGAGTAGCAACG	AGCTTGTCTAAGACGCCTGC
*GRIA4 short*	GATTCAAGATGTACCAACTCTTGGC	AAAATAGGATTCTTCATCAGAGGCA
*TFRC*	GGCACAGCTCTCCTATTGAAAC	CAAAGTCTCCAGCACTCCAACT

The transcript levels were quantified using the ΔΔCt method [19]. Samples showing Ct values higher than 45 were excluded.

### Protein expression analysis

**Fig. 1:**
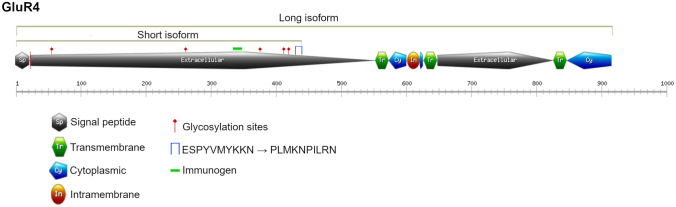
GluR4 protein isoforms and protein domains (green dash indicates the immunogen)

The protein expression levels of the long canonical and short GluR4 isoforms were studied by Western blot in a subgroup of ten matched tumour and normal tissues. Proteins from colon tissues were extracted using the Membrane Protein Extraction Kit (Mem-PERTM Plus, Thermo Fisher Scientific, Waltham, MA, USA) following the manufacturer’s protocol. Protein lysate separation was performed onto a 10% SDS–polyacrylamide gel at 100 V for 90 min. Then, protein transfer was done using 0.45 µm nitrocellulose membrane for 90 min. Afterwards, blots were blocked in 5% milk overnight at 4 °C and then incubated 2 h with primary antibody GluR4 (0.5 µg/mL, PA5-18931, Thermo Fisher Scientific, Waltham, MA, USA) and NaK ATPase (1:40,000, Ab76020, Abcam, Cambridge, UK) as gel-loading control. Once the hybridization time finished, the membrane was washed three times with Tris-buffer containing Tween-20, incubated with horseradish peroxidase-labelled secondary antibody (Jackson ImmunoResearch, Ely, UK) during 1 h at room temperature and washed again three times. Chemiluminescence was detected with ECL Chemiluminescent Western blotting reagents (Bio-Rad, Hercules, CA, USA). The intensity of Western blot signals was quantified using ImageJ programme and normalized respect to NaK ATPase. In Fig. 1 GluR4 protein isoforms and protein domains are illustrated, including the immunogen recognized by the antibody.

### Statistical analyses

Statistical differences in the quantitative levels between sample groups were evaluated considering the average ΔCt for methylation/mRNA and the band intensity for protein evaluation. Statistics was calculated using Welch’s *t* test. When statistical significant differences were detected in the expression level between groups, we considered upregulation or downregulation.

### miRNA targets prediction

We further evaluate post-transcriptional regulation by predicting miRNAs specific interactions with the two *GRIA4* transcripts studied in this work. To achieve this aim we used bioinformatics tools: TargetScan (http://www.targetscan.org/vert_72/), DIANA micro-T (http://diana.imis.athena-innovation.gr/DianaTools/) and miRDB (mirdb.org).

## Results

**Fig. 2:**
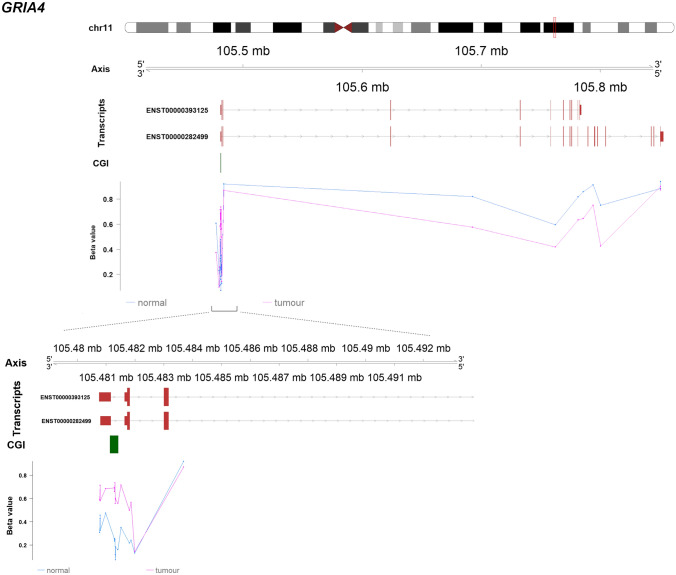
Genomic organisation of *GRIA4* including the localization of exons and CGI. Mean beta values resulting from the average of the samples (normal and tumour) of each probe mapping on the whole gene [1]. The zoom in focuses on the CGI located at the promoter region

As mentioned above, we previously detected hypermethylation of a CGI located in the promoter region of *GRIA4* [1] that may potentially affect the expression levels of the long and short isoforms. As shown in Fig. 2, normal cell DNA methylation pattern varies along *GRIA4* gene, with lower methylation values in the promoter region than in the gene body, while, in tumour samples, higher DNA methylation levels in the promoter region and lower in the gene body have been detected compared to controls.

### *GRIA4* methylation study

**Fig. 3:**
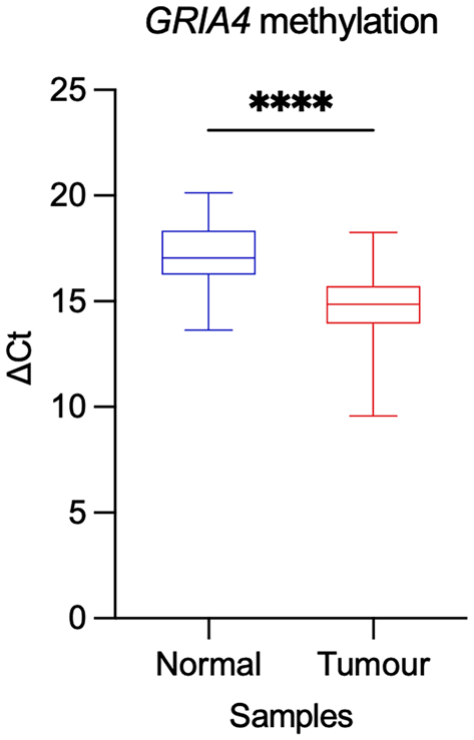
*GRIA4* methylation analysis. Box plot showing ΔCt values of *GRIA4* methylation for normal and tumour tissues comparison. Asterisks indicate statistically significant differences (****p value < 0.0001)

*GRIA4* methylation analysis of the investigation cohort confirmed a statistically significant hypermethylation (average ΔΔCt = − 2.33, *p* value < 0.0001) in tumoural samples compared to controls (Fig. 3), being ΔCt inversely correlated to methylation levels.

### mRNA expression study

**Fig. 4:**
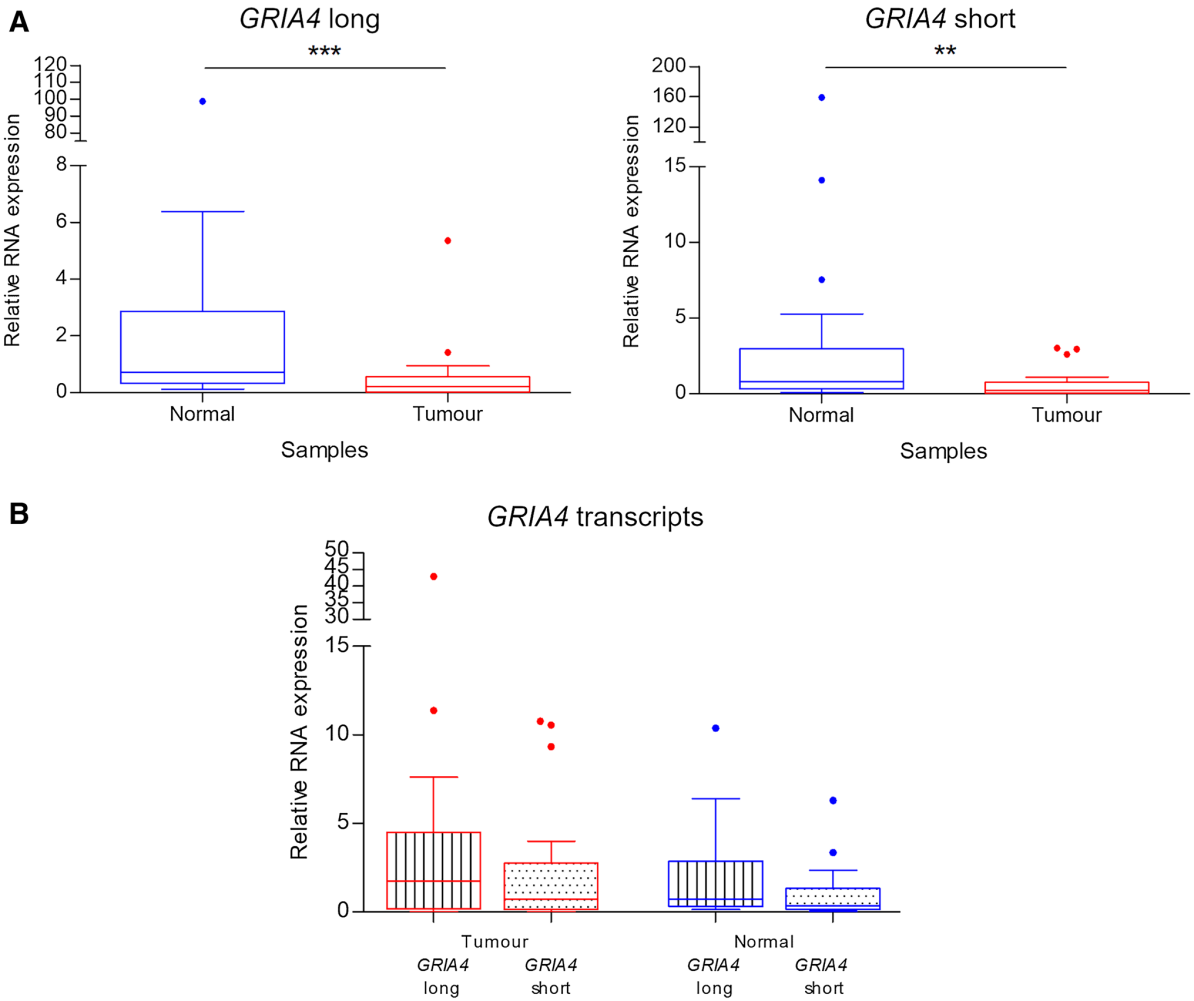
*GRIA4* gene expression analysis. **A** Box plot fold change values of *GRIA4* long (on the left) and short (on the right) for normal and tumour tissues comparison. **B** Box plot fold change values of *GRIA4* variants comparison within tumours and normal colon tissues. Asterisks indicate statistically significant differences (**p value < 0.01, ***p value < 0.001)

To evaluate whether hypermethylation in the promoter region of *GRIA4* affects the expression of both alternative transcripts, a gene expression analysis has been performed by qRT-PCR. As observed in Fig. 4 the two transcript variants of *GRIA4*, analysed in the current work, are expressed in tumour and normal tissues. However, there exists a statistically significant reduction of both transcripts in CRC samples respect to normal tissues (Fig. 4A). We compared the expression levels between alternative transcripts in each tissue type and no significant differences have been detected (Fig. 4B).

### Protein expression study

**Fig. 5:**
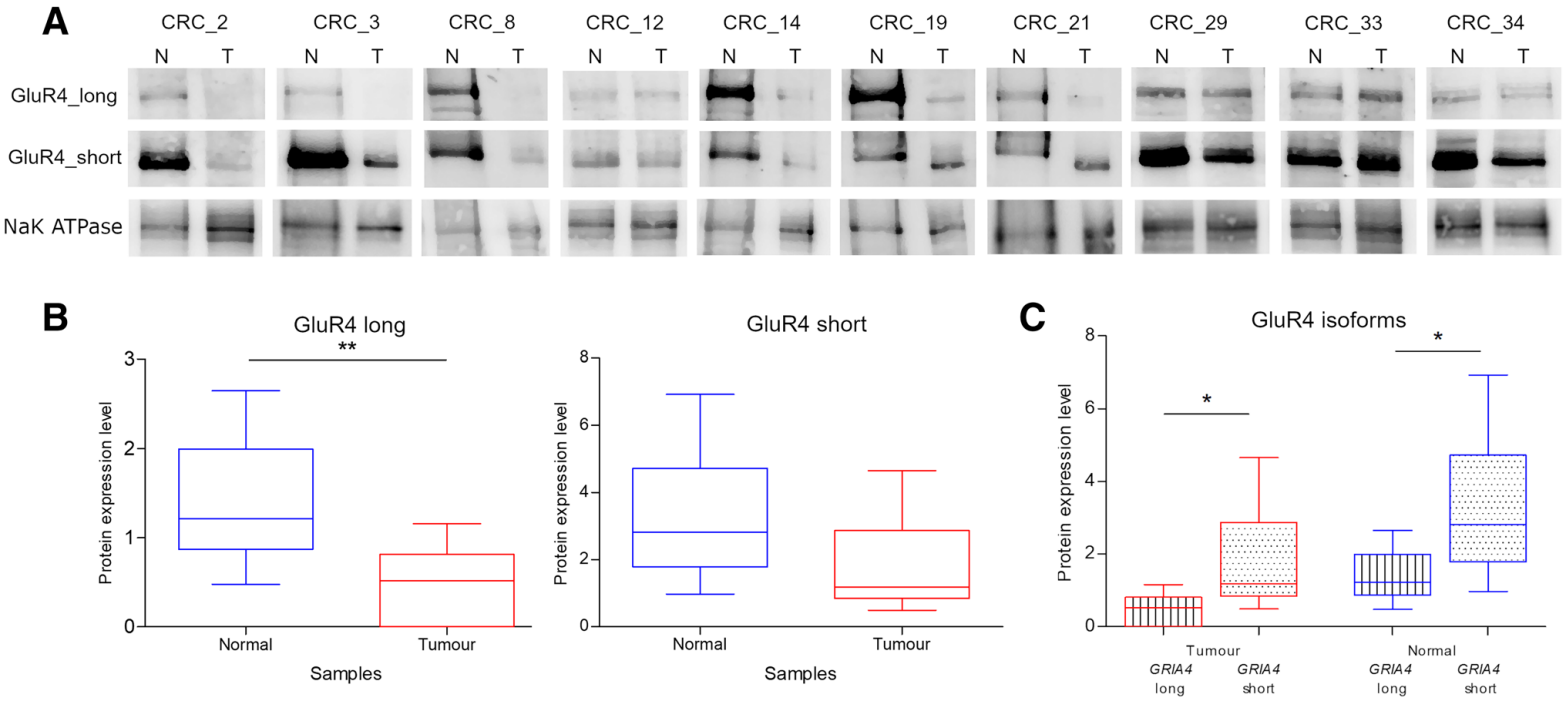
GluR4 protein expression analysis. **A** Representative blots of GluR4 isoforms in ten CRC paired tissue samples. NaK ATPase was used as loading control. **B** Box plots of GluR4 long and short expression in normal vs tumour samples. **C** Box plots of GluR4 isoforms within tumour and normal samples groups. Asterisks indicate statistically significant differences (*p value < 0.05, **p value < 0.01)

To study the protein expression, western blot analysis of GluR4 was performed in 10 paired samples analysed by qRT-PCR. Both isoforms of GluR4 were expressed in all samples, in agreement with their transcript expression (Fig. 5). A statistically significant reduction of GluR4 long was observed in CRC tissue, whereas the expression of the short isoform did not show any statistically significant difference between tumour and normal samples although a trend towards downregulation in the tumours can be observed (Fig. 5A and B). However, in contrast to the mRNA expression pattern of the two transcript variants, the short isoform of GluR4 displayed significant higher protein expression than the long isoform either in normal and tumoural tissues (Fig. 5C).

### miRNA targets analysis

To explain the opposite expression results between mRNA and protein, we further investigate possible post-transcriptional regulators, such as miRNAs, using bioinformatics tools. Interestingly, using TargetScan four miRNAs, miR-506-3p, miR-124-3p.1, miR-124-3p.2 and miR-137, highly conserved among vertebrates, with the strictest matching site types, *i.e.* branch length score 8mer and 7mer-A1, and high preferentially conserved targeting scores have been identified to target only the long *GRIA4* transcript. Table 4 summarizes the characteristics of the selected miRNAs, including the Context++ score that considers 14 features such as stability, conservation, TA abundance among others, and *P*_CT_ parameter that estimates the site conservation due to miRNA target selectivity. These interactions have been also reported by other prediction tools; DIANA micro-T showing high confident miTG score 0.99 and 0.92 for miR-124 and miR-506 respectively and miRDB predicting interactions with target scores of 90 for miR-506 and miR-124, and 80 for miR-137. Instead, we did not identify any miRNA-*GRIA4* short interaction using the same parameters.

**Table 4:**
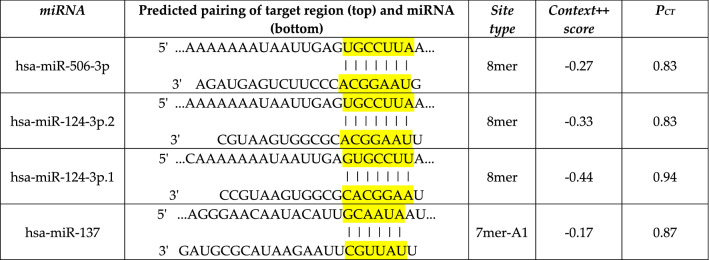
*GRIA4* long targeting miRNAs

## Discussion

Hypermethylation of *GRIA4*-associated promoter CGI is an early methylation event associated with *GRIA4* downregulation at mRNA and protein level [1, 3, 20, 21]. In the present work, we firstly verified, and confirmed, the hypermethylated status of the aforementioned CGI associated to *GRIA4* and further analysed its alternative transcripts and isoform expression levels in CRC paired tissue samples.

Several *GRIA4* transcript variants and two protein isoforms were described but their expression in colon has not yet been tested. These glutamate receptors have a pivotal function in the nervous system where they are highly expressed. The majority of *GRIA4* variants analyses are focused on the brain [22–25]. On the other hand, even the canonical isoform is lowly expressed in colon tissues, resulting in a possible underestimation of its involvement in cell intestinal biology (https://gtexportal.org/home/). However, an increasing number of evidences point out that genes involved in cellular crosstalk and in particular related to the nervous system are epigenetically altered in CRC [1, 26]. We analysed the expression levels of two *GRIA4* variants, long and short, that encode the canonical and the short isoforms. The novelty of our work resides on their detection for the first time using qPCR and Western blot in tumoural and normal colon tissues. The evident expression of both isoforms in a non-neuronal tissue opens a new research scenario to elucidate the glutamatergic signalling pathway in colon tissue.

As expected, the two variants were lower expressed in tumour tissue than in normal colon tissue, both at mRNA and protein levels.

The similar expression profile observed between alternative transcripts suggests that DNA hypermethylation regulate in the same way their transcription.

Opposite results were observed at protein level, where the short isoform resulted more expressed than the canonical isoform, both in normal and tumour tissues. This could be related, on one hand, to the lifetime of each isoform. In particular, the degradation mechanism of GluR4 is not well elucidated but previous evidence reported that caspases cleave GluR4 at the C-terminal domain contributing to selective proteolysis [27, 28]. This process could increase the susceptibility of GluR4 long canonical isoform to degradation but would not target the short one that lacks the C-terminal region. On the other hand, post-transcriptional regulation can be mediated by microRNAs (miRNAs). Four highly conserved miRNAs that target the long *GRIA4* transcript have been identified using TargetScan. In contrast, no interaction between miRNAs and *GRIA4* short transcript variant was predicted using the same parameters, suggesting a specific transcripts regulation and possibly resulting in the higher expression of the short isoform. The relationship between alternative transcripts and miRNA regulation is not well elucidated but previous works report variant-specific miRNA targeting [29–31].

Interestingly, although the unknown role of the short isoform, its constant expression in both tumoural and normal tissues led to expect that it may participate in cell function. It has been reported that the AMPA receptor assembly occurs in the endoplasmic reticulum through interactions between domains [15] and thus the extracellular domains of the short isoform could interact with the other glutamate receptor subunits. The extracellular region contains the signal peptide, also included in the short isoform that targets the protein to the membrane [15].

To conclude, our study is the first one to show the expression of different *GRIA4* isoforms in CRC and normal colon tissue. As expected by the promoter hypermethylation found in *GRIA4*, both alternative transcripts and isoforms are downregulated in CRC.

The evident but even higher expression of the short isoform compared to the long in both tissue samples may suggest a role in the intestinal cell biology. Functional studies are necessary to confirm this hypothesis. The expression of the short isoform may lead to effects in downstream signalling pathways. Glutamate receptors’ variants may modify the assembly and biophysical properties of the channels, affecting their main function as excitatory neurotransmitter receptors and their involvement in cellular homeostasis and in proliferation pathways such as the MAPK and PI3K/Akt, among others [32–35].

Our findings highlight the importance of post-transcriptional regulation mechanisms in *GRIA4* alternative isoforms expression. The biological processes responsible of their different half-life must be addressed in future experimental assays.

## Data Availability

The data that support the findings of this study are available from the corresponding author, P.Za., upon reasonable request.
